# Comparison between the long-axis in-plane and short-axis out-of-plane approaches for ultrasound-guided arterial cannulation: a meta-analysis and systematic review

**DOI:** 10.1186/s12871-023-02076-2

**Published:** 2023-04-13

**Authors:** Lei Cao, Yu-ting Tan, Ting Wei, Hong Li

**Affiliations:** Department of Anesthesiology, Second Affiliated Hospital of Army Medical University, Chongqing, China

**Keywords:** Ultrasound-guided, Long-axis in-plane, Short-axis out-of-plane, Arterial cannulation, Meta-analysis

## Abstract

**Background:**

The two most common methods for ultrasound-guided arterial cannulation are the long-axis in-plane (LA-IP) and short-axis out-of-plane (SA-OOP) approaches. However, it is uncertain which method is more advantageous. We conducted a meta-analysis of reported randomized clinical trials (RCTs) comparing the two techniques in terms of success rate, cannulation time, and complications.

**Methods:**

We systematically searched PubMed, Embase, and the Cochrane Library database for RCTs comparing the LA-IP and SA-OOP techniques for ultrasound-guided arterial cannulation published from inception through April 31, 2022. The Cochrane Collaboration’s Risk of Bias Tool was used to evaluate the methodological quality of each RCT. Review Manager 5.4 and Stata/SE 17.0 were used to analyze the two primary outcome measures (first-attempt success rate and total success rate) and two secondary outcome measures (cannulation time and complications).

**Results:**

A total of 13 RCTs with 1,377 patients were included. There were no significant differences in first-attempt success rate (risk ratio [RR], 0.93; 95% confidence interval [CI], 0.78–1.12; *P* = 0.45; I^2^ = 84%) and overall success rate (RR, 0.99; 95% CI, 0.95–1.02; *P* = 0.48; I^2^ = 57%). When compared with the LA-IP technique, the SA-OOP technique was associated with an increased incidence of posterior wall puncture (RR, 3.01; 95% CI, 1.27–7.14; *P* = 0.01; I^2^ = 79%) and hematoma (RR, 2.15; 95% CI, 1.05–4.37; *P* = 0.04; I^2^ = 63%). There was no significant difference in the incidence of vasospasm between techniques (RR, 1.26; 95% CI, 0.37–4.23; *P* = 0.07; I^2^ = 53%).

**Conclusions:**

The present results suggest that the SA-OOP technique is associated with a higher incidence of posterior wall puncture and hematoma than the LA-IP technique, whereas success rates are similar for the two ultrasound-guided arterial cannulation techniques. These findings should be experimentally evaluated in a more rigorous manner due to high inter-RCT heterogeneity.

## Introduction

Arterial puncture is a common procedure in surgical settings, intensive care units, and emergency departments [1–3], allowing for ambulatory blood pressure monitoring and blood gas analysis. Although arterial cannulation can be performed at various sites, the radial artery is often the preferred site because of its shallow location, the adequate blood supply to the side branches, low complication rates, and ease of operation [4, 5]. In most cases, traditional arterial cannulation is performed using palpation, which is challenging and can be more difficult in children, infants, and some critically ill patients. Furthermore, repeated cannulation attempts may lead to complications such as hematoma, thrombosis, infection, and nerve injury [4]. In recent years, ultrasound-guided arterial puncture has been increasingly used in clinical practice. Numerous studies have demonstrated that ultrasound-guided arterial cannulation is associated with higher success rates and lower complication rates than traditional arterial cannulation in both children and adults [3, 6–8].

The two most common methods for ultrasound-guided arterial cannulation are long-axis in-plane (LA-IP) and short-axis out-of-plane (SA-OOP) approaches [9–11]. In the SA-OOP approach, the target vessel is visualized in relation to the adjacent tissue, and the visible portion of the needle is in the center of the vessel. However, the ultrasound plane may pass proximally through the needle axis, leading to an underestimation of the depth of the needle tip [10]. In contrast, the LA-IP approach provides a better indication of the length of the puncture needle and its position relative to the posterior wall of the artery [12]. Nonetheless, maintaining the alignment of the ultrasound plane with the vessel may be more difficult using the LA-IP approach because the image plane may deviate to one side of the needle plane [13]. However, it is uncertain which method is more advantageous [14, 15].

Therefore, in the present study, we aimed to conduct a meta-analysis of reported randomized clinical trials (RCTs) that investigated the performance of the SA-OOP and LA-IP techniques in arterial cannulation in terms of success rate, cannulation time, and complications.

## Methods

The current meta-analysis was performed in accordance with the Preferred Reporting Items for Systematic Review and Meta-analysis (PRISMA) [16], and the protocol for the systematic review was registered at PROSPERO on May 10, 2022 (registration ID: CRD42022321504). We systematically searched PubMed, EMBASE, and the Cochrane database for articles containing the following Chinese and English keywords published from inception to April 31, 2022: “ultrasound” “ultrasonography,” “ultrasonic,” AND “catheterization,” “cannulation,” AND “long axis in plane,” “short axis out of plane,” “long axis,” “short axis,” “in plane,” “out of plane,” AND “RCTs.” We tried to get missing data by contacting the original author by email. Each retrieved reference was screened to determine whether it met the inclusion criteria. The included trials and relevant comments are listed in Tables 1 and 2. The inclusion criteria were as follows: arterial cannulation performed in all patients; comparison of the two techniques for arterial cannulation; publication in English; and randomized controlled design (i.e., RCTs).

**Table 1:** Characteristics of the trials

Author/year	Country	Patients	Artery	No. of patients(M/F)	Operators	Cannula	Age(S/L)	Weight(S/L)
Abdalla et al. 2017 [17]	Egypt	adult	RA	S:42 L:42	Expert operators	ND	S: 55 ± 11 L: 59 ± 9	(84 ± 32)/(84 ± 31)(kg)
Abdelbaser et al. 2022 [18]	Egypt	Neonates/ infants	FA	S:41(20/21) L:41(20/21)	Pediatric cardiac anesthesiologist	24-G	S: 127.6 ± 37.9(d) L: 135.4 ± 36.5(d)	ND
Arora et al. 2020 [19]	Oman	adult	RA	S:42 L:42	Eperienced anaesthesiologist	20-G	S: 54.1 ± 17.17 L: 56.69 ± 14.82	BMI:(26.89 ± 4.22)/(26.98 ± 4.17)
Berk et al. 2013 [14]	Turkey	adult	RA	S:54(23/31) L:54(30/24)	Two staff anesthetists (more than 50 arterial lines)	20-G	S: 56 ± 1 L: 54 ± 2	(78 ± 18)/(76 ± 16)(kg)
Boran et al. 2020 [20]	Turkey	newborns	FA	S:34(14/20) L:31(16/15)	Clinician with approximately 20 years of experience	24-G	S: 17.2 ± 7.4(d) L: 17.16 ± 7.04(d)	(3.18 ± 0.9)/(3.13 ± 0.85)(kg)
Cao et al. 2021 [21]	China	adult	RA	S:70(32/38) L:63(34/29)	Anaesthesia residents	22-G	S: 50 ± 12 L: 52 ± 10	BMI:(25 ± 4)/(25 ± 4)
Nam et al. 2020 [22]	Korea	adult	RA	S:70(43/27) L:66(36/30)	Single operator (more than 100cases of RA cannulation per year)	20-G	S: 64.3 ± 13 L: 63.6 ± 13.3	(64.3 ± 14.9)/(63.2 ± 12.2)(kg)
Quan et al. 2014 [15]	China	adult	RA	S:81(59/22) L:82(64/18)	The same experienced anaesthesiologist	ND	S: 49.2 ± 8.1 L: 46.1 ± 7.9	(76.4 ± 12.2)/(72.1 ± 10.5)(kg)
Rajasekar et al. 2022 [23]	India	adult	RA	S:30(20/10) L:30(20/10)	The same experienced anaesthesiologist	20G	ND	ND
Sethi et al. 2016 [24]	India	adult	RA	S:75(46/29) L:75(41/34)	Two anesthetists (more than 100 arterial lines)	20-G	S: 59.5 ± 8.2 L: 57.7 ± 7.6	(62.8 ± 11.6)/(64.6 ± 12.2)(kg)
Song et al. 2016 [25]	Korea	infants/children	RA	S:50(37/13) L:51(35/16)	Two paediatric anaesthesiologists (more than 20 arterial cannulations and 200 central venous catheterisations)	24-G	Infants S: 5.6 ± 3.7(m) L: 4.3 ± 3.0(m) Children S: 3.4 ± 2.8(y) L: 3.3 ± 1.2(y)	Infants (7.4 ± 2.1)/(6.8 ± 2.3) Children (14.4 ± 3.3)/(14.8 ± 3.9)
Wang et al. 2022 [26]	China	adult	RA	S:65(16/49) L:66(15/51)	Four anaesthesiologists (more than 160 arterial cannulations)	22-G	S:67.5 ± 17.8 L:67.5 ± 13.8	BMI:(21.9 ± 3.5)/(22.2 ± 3.5)
Yu et al. 2022 [27]	China	children	RA	S:40(25/15) L:40(21/19)	The same experienced anesthetists (more than 100 arterial cannulations)	24- or 22-G	S:2.7 ± 0.7 L:2.7 ± 0.9	(13.1 ± 1.9)/(13.7 ± 2.2)(kg)

*RA* Radial artery, *FA* Femoral artery, *S* Short-axis out-of-plane, *L* Long-axis in-plane, *ND* Not describe, *BMI* Body mass index

**Table 2:** Characteristics of the trials

Author/year	Ultrasound system	Clinical setting	Primary outcomes
Abdalla et al. 2017 [17]	Toshiba Xario, Japan, PLT 805AT transducer; 8 MHz; depth, 3 cm	operative and ICU patients	Overall success rate
Abdelbaser et al. 2022 [18]	General Electric Ving Med Systems, Horten, Norway	elective cardiac surgery	The rate of a successful first puncture
Arora et al. 2020 [19]	Philips Ultrasound, Bothell, WA;15 megahertz [range 7–15]	CABG	The number of first-pass successful attempts
Berk et al. 2013 [14]	Esaote My Lab 30, US Machine, Florance, Italy; 18 MHz, 2 cm depth	elective surgery	Cannulation time
Boran et al. 2020 [20]	NextGen LOGIQ e Ultrasound GE Healthcare (China) Co. Ltd; 4–12 MHz	NICU patients requiring arterial monitoring	Success rate
Cao et al. 2021 [21]	SONIMAGE HS1, KONICA MINOLTA, China; 5 to 13 MHz	elective surgery	The rate of success
Nam et al. 2020 [22]	iE33, Philips; L15-7io	cardiac surgery	The first-attempt success rate of cannulation
Quan et al. 2014 [15]	Terason2000 + , Terason, Burlington, MA; 18 MHz, 2 cm depth	liver surgery or splenic resection	The first-attempt success rate of cannulation
Rajasekar et al. 2022 [23]	Sonosite R ultrasound system, Sonosite Inc., Bothell, WA, USA	elective surgery	The first-attempt success rate
Sethi et al. 2016 [24]	Sonosite® MicroMaxx® Ultrasound System, Sonosite INC, Bothell, WA, USA; 13–6 MHz	elective surgery (cardiothoracic, general, orthopaedic or neurosurgery)	Cannulation characteristics
Song et al. 2016 [25]	LOGIQ e; GE Healthcare, Wauwatosa, Wiscon- sin, USA; 4–10 MHz	elective surgery	The total time to successful cannulation
Wang et al. 2022 [26]	SonoSite M-Turbo Color Doppler Ultrasound Diagnostic instrument; L25 N/13–6	elective surgery, RA diameters < 2.2 mm	The cannulation success rate
Yu et al. 2022 [27]	Huasheng Medical Company, China, 15L linear array probe; 5–13 MHz, depth 3 cm	elective surgery	The first-attempt cannulation success rate

*ICU* Intensive care unit, *CABG* Coronary bypass surgery, *NICU* A neonatal intensive care unit

### Trail selection

**Fig. 1:**
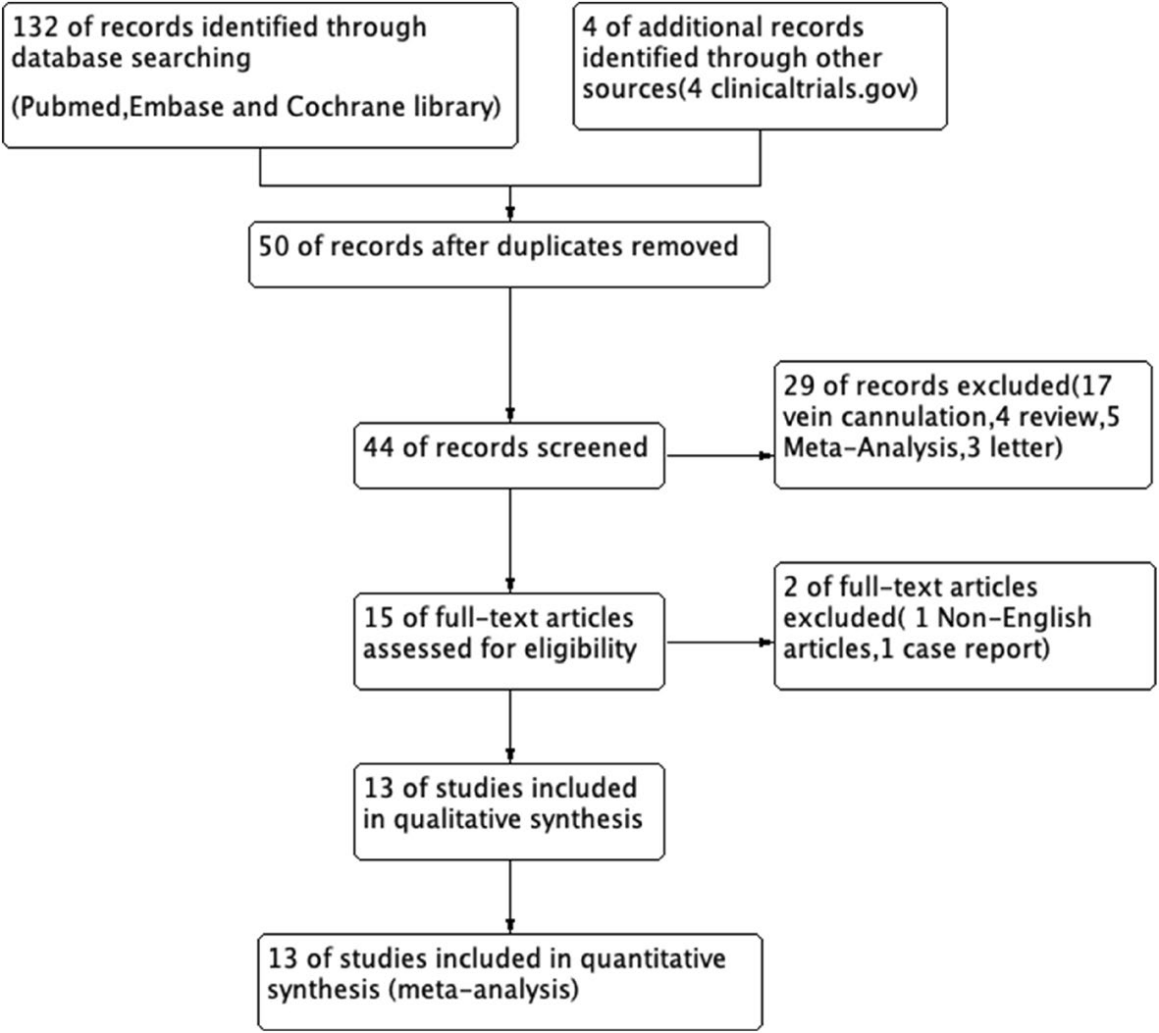
Flowchart of study selection

Two authors (L.C and Y.T.T) independently screened the retrieved trials against the inclusion criteria. The decision to include a retrieved trial was made only when the two authors reached an agreement. Disagreements between the two researchers were resolved through discussion, and when an agreement could not be reached, the opinion of a third author (H.L.) was sought. In the case of incomplete or missing data, the original authors of the trial were contacted via email to obtain the data. The results of the trial selection process are presented in the PRISMA flowchart (Fig. 1).

### Study characteristics and data extraction

For each included trial, information concerning the following features was collected, including lead author’s name, country, trial year, patient group, number of patients, operators, puncture site, type of puncture needle, patient age, type of surgery, type of equipment, and primary outcome (Tables 1 and 2). Data were independently extracted by two authors (L.C and Y.T.T), and inconsistencies were resolved through discussion and review. First-attempt success rate and overall success rate were selected as the primary outcome measures, while cannulation time and complications were selected as the secondary outcome measures.

### Risk-of-bias assessment

**Fig. 2:**
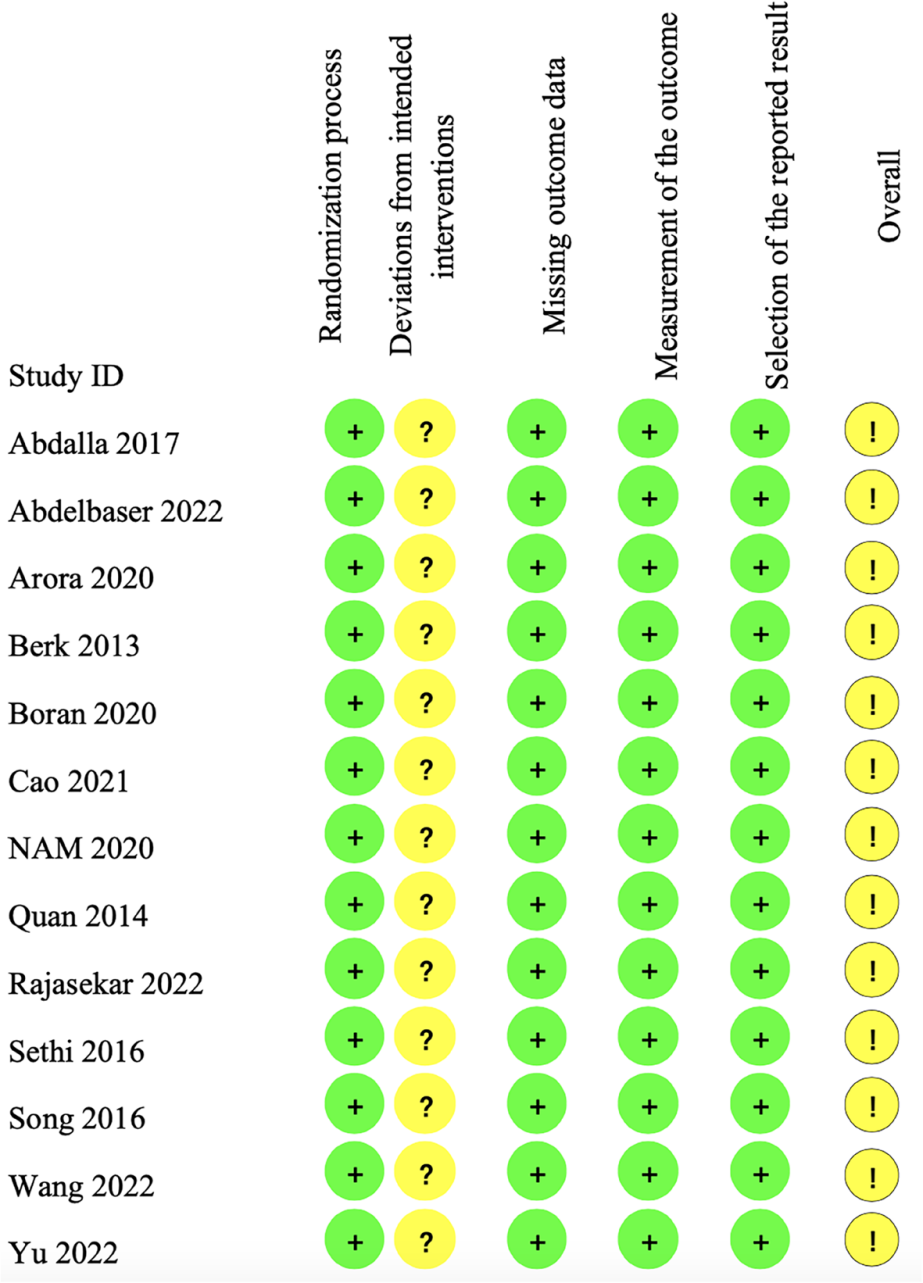
Cochrane Risk of Bias 2.0 Tool included randomized controlled trials. The green circle indicates low risk of bias, yellow circle indicates some concerns risk of bias

The quality of each RCT was assessed separately by two authors (L.C. and Y.T.T.) using the Cochrane Risk of Bias 2.0 tool for RCTs [28] in terms of the randomization process, deviations from intended interventions, missing outcome data, measurement of the outcome, and selection of the reported results, disagreements were resolved by discussion with a third investigator (H. L.). The RoB for each of the 5 domains and overall was described as low, some concerns, or high. The results of the risk-of-bias assessment are detailed in Fig. 2.

### Statistical analysis

Data analysis was performed using Review Manager 5.4 (Cochrane Collaboration, Oxford, UK) and Stata/SE 17.0 software (StataCorp, College Station, TX). Some of the data were expressed as quartiles and medians with standard deviations using the estimation methods reported by Luo et al. [29] and Wan et al. [30]. Continuous data (cannulation time) were expressed as the mean difference or standardized mean difference and 95% confidence interval (CI). Dichotomous data (success rate, complications) were expressed as risk ratios (RRs) with 95% CIs. The degree of heterogeneity between RCTs was assessed using the χ^2^ test (P-value and I^2^-value). The random-effects model was adopted for cases of inter-RCT heterogeneity (*P* ≤ 0.05 or I^2^ > 50%), while the fixed-effects model was adopted for other cases (*P* > 0.05 or I^2^ ≤ 50%). Sensitivity analysis was performed to identify the causes of significant inter-RCT heterogeneity. Subgroup analysis was performed to examine the impacts of the patient group, age, and trial operator on both methods.

We used the Grading of Recommendations, Assessment, Development, and Evaluation (GRADE) approach to classify the certainty of evidence into high, moderate, low, or very low for each outcome [31].

## Results

### Study selection and characteristics

**Fig. 3:**
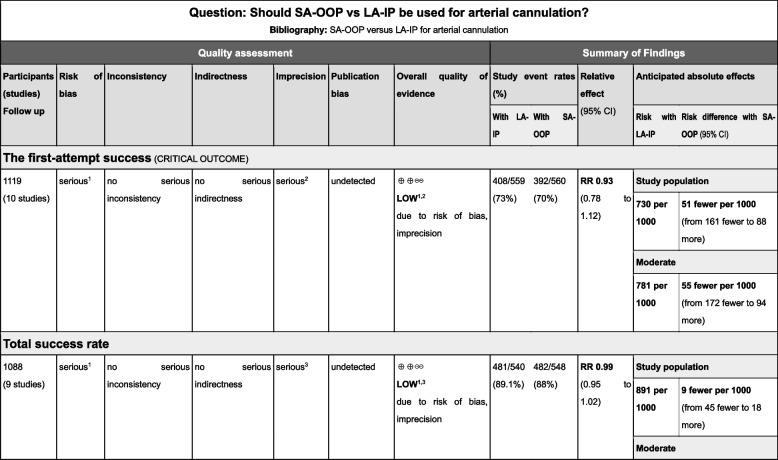
Summary of findings

Thirteen RCTs [14, 15, 17–27] with a total of 1,377 patients were included in the meta-analysis (Fig. 1). The patient characteristics, interventions, and primary outcome indicators of each RCT are summarized in Tables 1 and 2. All trials compared the safety and efficacy of both techniques for arterial cannulation and reported success rates, cannulation time, and complications for both techniques. We present our assessment of the certainty of evidence for each outcome according to the GRADE approach in the summary of findings table (Fig. 3).

### First-attempt success rate

**Fig. 4:**
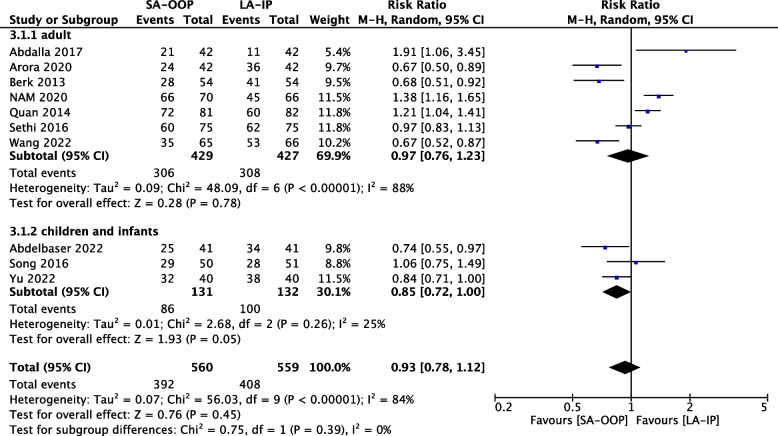
The first-attempt success rate between SA-OOP and LA-IP

Ten of the 13 included studies (*n* = 1,119) reported first-attempt success rates for both arterial cannulation techniques [15, 17–19, 21–24, 26, 27]. There were no significant differences in first-attempt success rate between the two techniques (RR, 0.93; 95% CI, 0.78–1.12; *P* = 0.45; I^2^ = 84%) (Fig. 4). Although the first-attempt success rate of LA-IP was higher than that of SA-OOP but the difference between the two techniques was not statistically significant (RR, 0.93; 95% CI, 0.78–1.12; *P* = 0.45; I2 = 84%). The risk ratio was 0.93 with a 95% confidence interval of 0.78 to 1.12, *P* value 0.45, I^2^ = 84% (Fig. 4).

### Total success rate and cannulation time

**Fig. 5:**
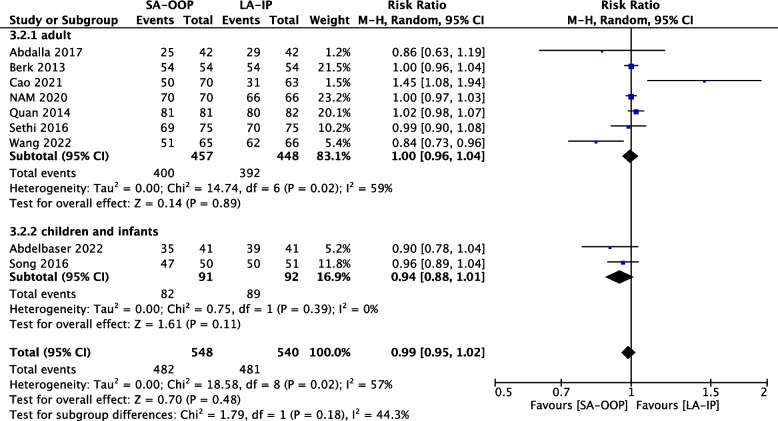
The total success rate between SA-OOP and LA-IP

Nine (*n* = 1,088) RCTs reported total success rates for both arterial cannulation techniques [14, 15, 17, 18, 21, 22, 24–26] and all included studies reported the cannulation time (*n* = 1,377) [14, 15, 17–27]. There were no significant differences in total success rate (RR, 0.99; 95% CI, 0.95–1.02; *P* = 0.48; I^2^ = 57%) (Fig. 5) between the SA-OOP and LA-IP techniques. The difference in total success rate between the SA-OOP and LA-IP techniques was not statistically significant (RR, 0.99; 95% CI, 0.95–1.02; *P* = 0.48; I2 = 57%) (Fig. 5). The risk ratio was 0.99 with a 95% confidence interval of 0.95 to 1.02, *P* value 0.48, I^2^ = 57% (Fig. 5). And all included studies reported the cannulation time (*n* = 1,377) [14, 15, 17–27]. As there were different definitions of cannulation time for each trial, we could not combine the puncture times for the analysis, and very different results emerged for each trial.

### Complications

**Fig. 6:**
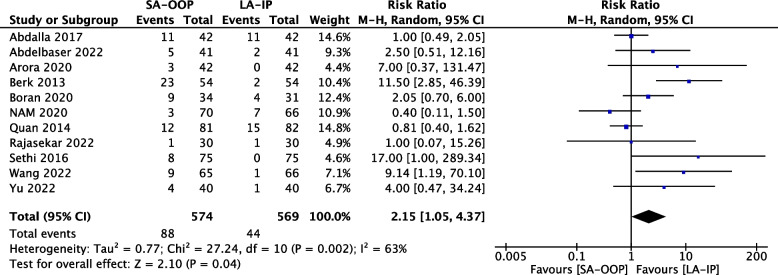
The rate of hematoma between SA-OOP and LA-IP

**Fig. 7:**
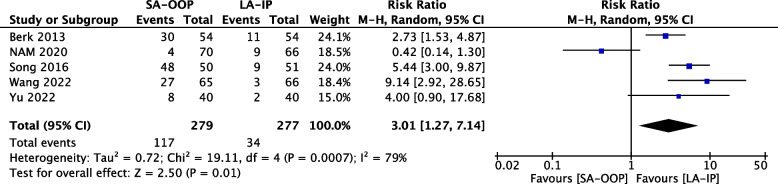
The rate of posterior wall puncture between SA-OOP and LA-IP

**Fig. 8:**
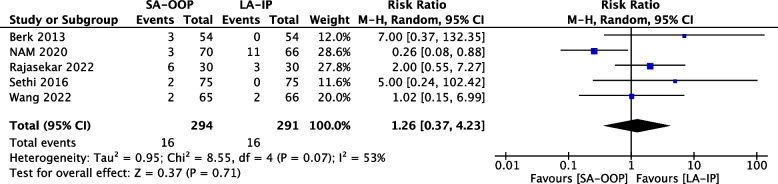
The rate of vasospasm between SA-OOP and LA-IP

**Fig. 9:**
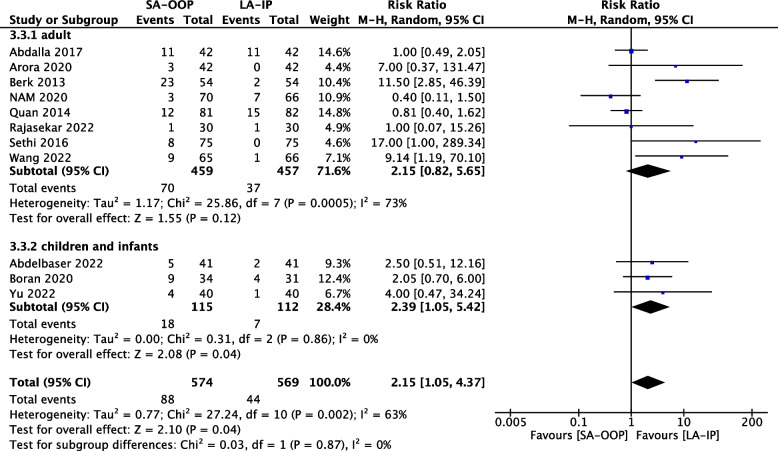
Subgroup analysis of the rate of hematoma by type of participant using a random effects model

**Fig. 10:**
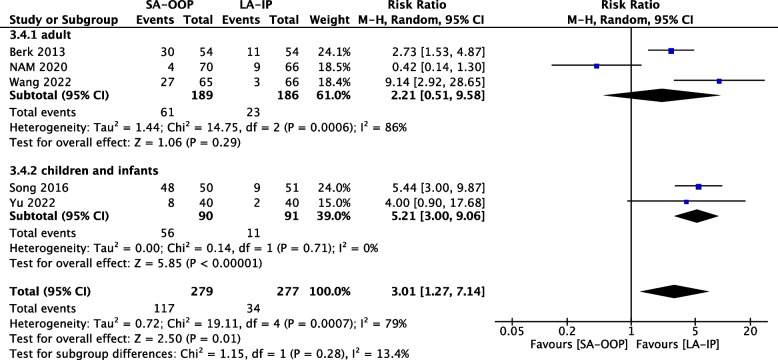
Subgroup analysis of the rate of posterior wall puncture by type of participant using a random effects model

Eleven (*n* = 1,143) trials reported the incidence of hematoma [14, 15, 17–20, 22–24, 26, 27] and five (*n* = 556) trials reported the incidence of posterior wall puncture [14, 22, 25–27]. Our analysis revealed lower incidences of hematoma (RR, 2.15; 95% CI, 1.05–4.37; *P* = 0.04; I^2^ = 63%) (Fig. 6) and posterior wall puncture (RR, 3.01; 95% CI, 1.27–7.14; *P* = 0.01; I^2^ = 79%) (Fig. 7) with the LA-IP technique than the SA-OOP technique. Five (*n* = 585) trials reported incidence of vasospasm [14, 22–24, 26], which did not differ significantly between the LA-IP and SA-OOP techniques (RR, 1.26; 95% CI, 0.37–4.23; *P* = 0.71; I^2^ = 53%) (Fig. 8). Subgroup analysis showed that the SA-OOP technique had higher rates of posterior wall puncture and hematoma in the pediatric subgroup, but in the adult subgroup, there were no statistical differences between the two techniques and no inter-subgroup differences (Figs. 9 and 10).

### Meta-analysis

**Fig. 11:**
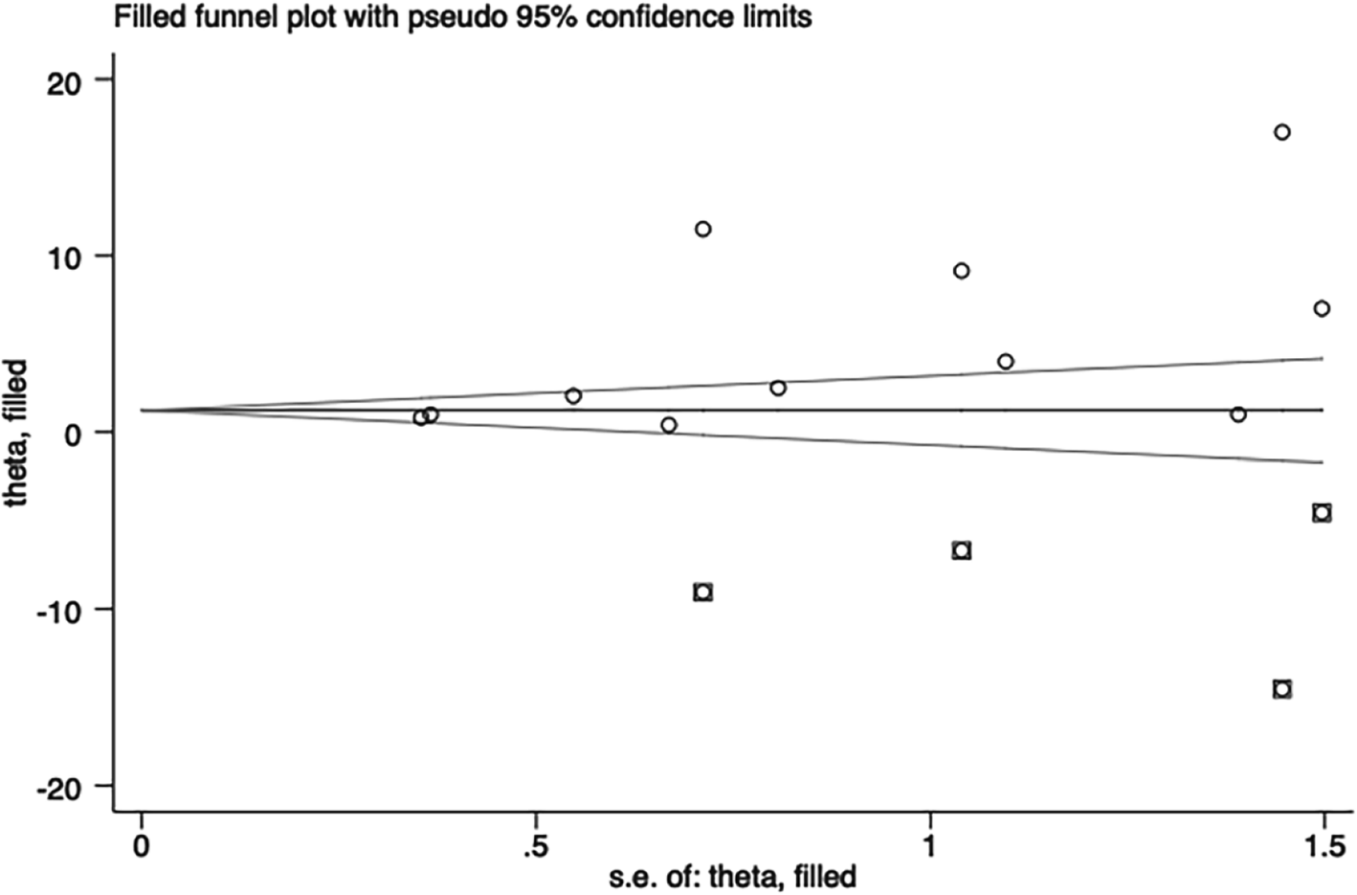
The trim and fill method for hematoma. The software estimated the number of missing to be 4

**Fig. 12:**
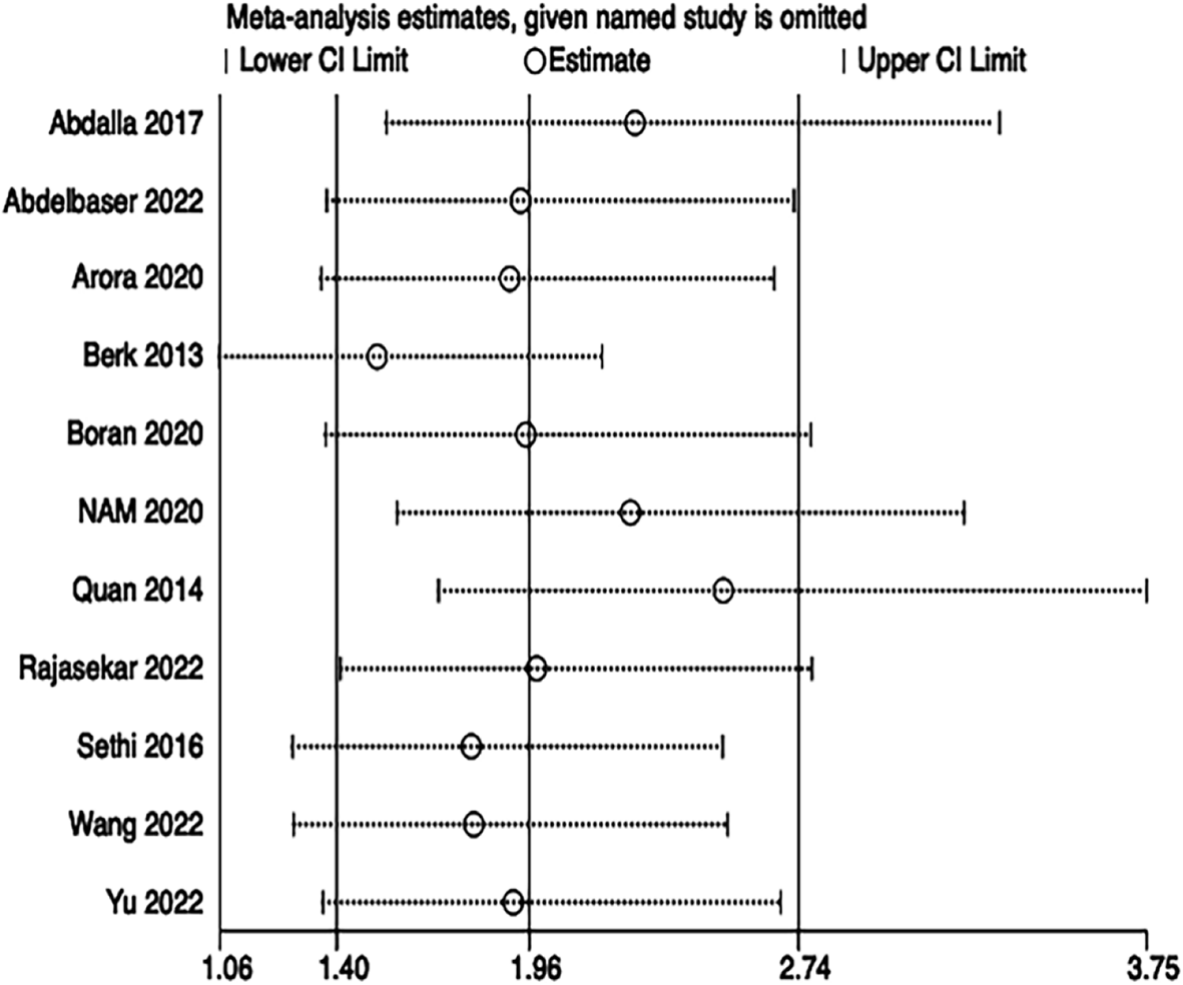
The plot of sensitivity analysis of hematoma

**Fig. 13:**
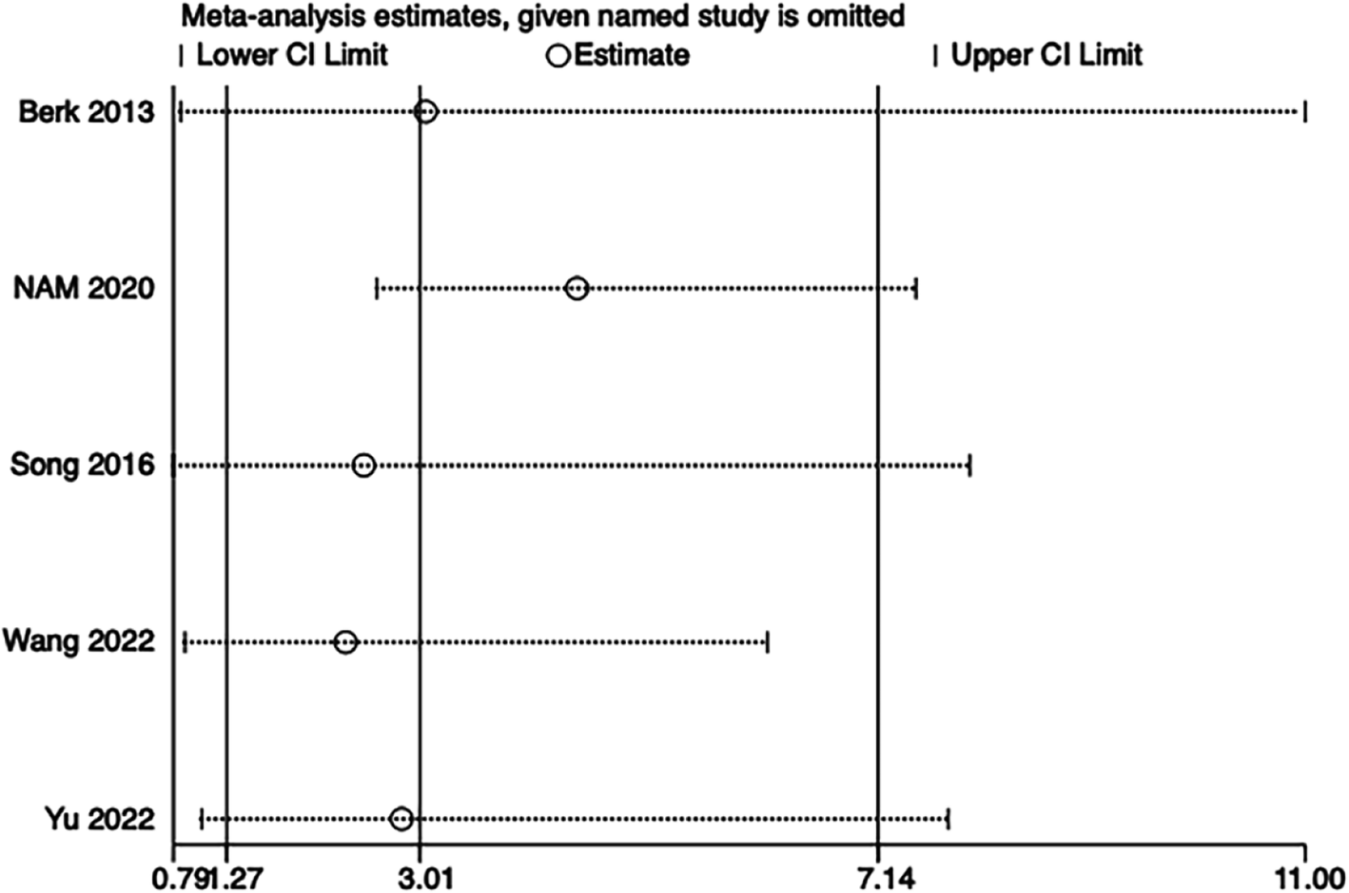
The plot of sensitivity analysis of vasospasm

Egger’s regression test of first-attempt success rate, total success rate, cannulation time, posterior wall puncture, and vasospasm indicated little evidence of publication bias (Table 3). As implementing Egger tests for publication bias significantly altered the results (*P* = 0.038 for hematoma), the trim and fill method was adopted to adjust publication bias for hematoma (Fig. 11). Implementing sensitivity analysis for the current meta-analysis was also performed, indicating that the results were reliable and statistically stable (Figs. 12 and 13).

**Table 3:** Egger test of publication bias

Std_Eff	Coefficient	SE	t	*p* >|t|	(95% CI)
Bias (first-attempt success rate)	−1.88	2.33	−0.81	0.442	−7.26 to 3.49
Bias (total success rate)	−0.41	1.07	−0.38	0.719	−3.17 to 2.35
Bias (hematoma)	2.04	0.84	2.42	0.038	0.14 to 3.94
Bias (posterior wall puncture)	−1.13	3.11	−0.36	0.741	−11.02 to 8.76
Bias(vasospasm)	2.44	1.65	1.48	0.235	−2.81 to 7.69

## Discussion

The present meta-analysis comparing the SA-OOP and LA-IP techniques for ultrasound-guided arterial cannulation included 13 RCTs with 1,377 patients. The results indicated that the rates of posterior vessel wall damage and hematoma were lower for the LA-IP technique than the SA-OOP technique, although there were no significant differences in first-attempt success rate, total success rate, cannulation time, or rates of other complications between techniques. As there were different definitions of cannulation time for each trial, we could not combine the puncture times for the analysis, and very different results emerged for each trial.

Several studies have verified that ultrasound guidance improves the success rate of arterial cannulation and reduces the incidence of associated complications when compared with the traditional palpation approach [6, 32, 33]. Ultrasound-guided arterial cannulation is mainly achieved via two methods, LA-IP and SA-OOP, although some studies have reported the use of an oblique approach [17, 21]. Some RCTs have aimed to determine which method provides the greatest advantages. However, while some have reported that the LA-IP technique is associated with a significantly higher first-attempt success rate than the SA-OOP technique [14, 19, 26], others have reported the opposite finding [15, 22]. The present meta-analysis revealed no significant difference in first-attempt success rate between the two techniques, which is consistent with the results of another meta-analysis [34]. One study argued that success rates may be higher when the puncture needle is positioned at a 45° angle to the radial artery [35]. Given the large inter-RCT heterogeneity in first-attempt success rate, we performed a sensitivity analysis, which revealed no significant change in the overall RR for the success rate. This finding suggests that the observed heterogeneity occurred due to clinical and methodological differences, such as differences in operator experience, variations in vascular condition and weight among individual patients, and differences in ultrasound equipment. In addition, some procedures were performed under general anesthesia, while others were performed under local anesthesia. It remains uncertain whether different degrees of pain and tension under different anesthesia states have an impact on the surgical operation of target blood vessels, necessitating further research.

Studies have also reported discrepant findings concerning the relative cannulation times for each technique. Some studies have reported that cannulation times are longer for the SA-OOP technique than the LA-IP technique [14, 18], while others have reported the opposite [17, 22]. This inconsistency may be attributable to differences in ultrasound scan time and needle tip repositioning. In contrast, the present study revealed no significant difference in cannulation time between the two techniques. Therefore, inter-RCT heterogeneity may have occurred due to differences in operator experience, patient characteristics, and ultrasound type.

During arterial cannulation, the success of the first puncture attempt is important [36], as failed puncture may lead to vasospasm, hematoma, thrombosis, posterior wall puncture, nerve injury, or infection complications that may be overlooked in the absence of ultrasound evaluation [12, 32]. The reported probabilities of massive hemorrhage from the radial and femoral arteries are 0.05% and 1.58–2.3%, respectively [4, 37], suggesting that it is highly necessary to prevent puncture-induced hematoma. Some meta-analyses have reported no significant difference in the incidence of hematoma between the two techniques [33, 38]. In another study, however, the incidence of posterior wall puncture was higher for the SA-OOP technique than for the LA-IP technique [25], consistent with our findings. In the SA-OOP technique, a fixed image plane perpendicular to the vessel is generated, and the needle axis is often mistaken for the needle tip [10, 12]. In contrast, the LA-IP technique allows the operator to monitor the target artery and the needle tip throughout the procedure, reducing the likelihood of posterior wall puncture or needle tip misalignment. In addition, although vasospasm is more common in neonates and very young children than in adults [39], the present study revealed no significant difference in the incidence of vasospasm between the two techniques.

Several studies involving both models and human patients have noted large differences between the SA-OOP and LA-IP techniques based on the level of experience with arterial cannulation [21]. Ultrasound-guided arterial cannulation is highly dependent on the operator’s experience and should be practiced on animal models to ensure adequate anatomical knowledge and understanding of complications before application in human patients [11].

The present meta-analysis has several limitations. First, patients in the included RCTs were all hemodynamically stable, which prevented us from analyzing the differences between the two techniques in critically ill patients and those with shock or hypotension. Second, the included RCTs were highly heterogeneous in terms of operator experience, type of surgery, and ultrasound equipment, which may have introduced bias. Lastly, the requirement for a blinded setting in RCTs may have also contributed to the occurrence of bias.

## Conclusion

In summary, the results of the present meta-analysis indicate that the SA-OOP technique is associated with a higher incidence of posterior wall puncture and hematoma than the LA-IP technique, whereas success rates and cannulation times are similar between the two techniques. These findings should be experimentally evaluated in a more rigorous manner due to high inter-RCT heterogeneity.

## Data Availability

The datasets generated and analyzed during the current study are available from the corresponding author upon reasonable request.

## Abbreviations

LA-IP: Long-axis in-plane
SA-OOP: Short-axis out-of-plane
RCT: Randomized controlled trial
RR: Risk ratio
CI: Confidence interval
OR: Odds ratio
